# Body Mass Index and Kidney Stones: A Cohort Study of Japanese Men

**DOI:** 10.2188/jea.JE20150049

**Published:** 2016-03-05

**Authors:** Eiichi Yoshimura, Susumu S. Sawada, I-Min Lee, Yuko Gando, Masamitsu Kamada, Munehiro Matsushita, Ryoko Kawakami, Ryosuke Ando, Takashi Okamoto, Koji Tsukamoto, Motohiko Miyachi, Steven N. Blair

**Affiliations:** 1 Department of Food and Health Science, Prefectural University of Kumamoto, Kumamoto, Japan; 1 熊本県立大学環境共生学部; 2 Department of Nutritional Education, National Institute of Health and Nutrition, Tokyo, Japan; 2 国立研究開発法人医薬基盤・健康・栄養研究所 栄養教育研究部; 3 Department of Health Promotion and Exercise, National Institute of Health and Nutrition, Tokyo, Japan; 3 国立研究開発法人医薬基盤・健康・栄養研究所 健康増進研究部; 4 Department of Epidemiology, Harvard School of Public Health, Boston, MA, USA; 4 ハーバード大学医学大学院; 5 Division of Preventive Medicine, Department of Medicine, Brigham and Women’s Hospital and Harvard Medical School, Boston, MA, USA; 5 ハーバード大学公衆衛生大学院; 6 Graduate School of Sport Sciences, Waseda University, Saitama, Japan; 6 早稲田大学大学院 スポーツ科学研究科; 7 Department of Nephro-Urology, Nagoya City University Graduate School of Medical Sciences, Aichi, Japan; 7 名古屋市立大学大学院医学研究科; 8 Department of Safety and Health, Tokyo Gas Co. Ltd., Tokyo, Japan; 8 東京ガス株式会社安全健康・福利室; 9 Department of Exercise Science, Arnold School of Public Health, University of South Carolina, Columbia, SC, USA; 9 サウスカロライナ大学公衆衛生大学院運動科学科; 10 Department of Epidemiology and Biostatistics, Arnold School of Public Health, University of South Carolina, Columbia, SC, USA; 10 サウスカロライナ大学公衆衛生大学院疫学生物統計学科

**Keywords:** kidney stone, body mass index, cardiorespiratory fitness, Japanese men, 腎結石, BMI, 全身持久力, 日本人男性

## Abstract

**Background:**

In Japan, the incidence of kidney stones has increased markedly in recent decades. Major causes of kidney stones remain unclear, and limited data are available on the relationship between overweight/obesity and the incidence of kidney stones. We therefore evaluated body mass index (BMI) and the incidence of kidney stones in Japanese men.

**Methods:**

Of the workers at a gas company, 5984 males aged 20–40 years underwent a medical examination in 1985 (baseline). This study includes 4074 of the men, who were free of kidney stones at baseline and underwent a second medical examination performed between April 2004 and March 2005. BMI was calculated from measured height and weight in 1985, and men were categorized into tertiles. The development of kidney stones during follow-up was based on self-reports from questionnaires at the second medical examination.

**Results:**

The average duration of follow-up was 19 years, with 258 participants developing kidney stones during this period. Using the lowest BMI (1st tertile) group as a reference, the hazard ratios (95% confidence intervals [CIs]) for the 2nd and 3rd BMI tertiles were: 1.26 (95% CI, 0.92–1.73) and 1.44 (95% CI, 1.06–1.96), respectively (*P* for trend = 0.019). After additionally adjusting for potential confounders, such as age, systolic blood pressure, cardiorespiratory fitness, cigarette smoking, and alcohol consumption, the hazard ratios were 1.28 (95% CI, 0.93–1.76) and 1.41 (95% CI, 1.02–1.97), respectively (*P* for trend = 0.041).

**Conclusions:**

These results suggest that increased BMI is a risk factor for kidney stones in Japanese men.

## Abstract (ja)

**【背景】:**

近年、日本において腎結石罹患率は急激に増加している。腎結石の主要な原因は明確になっておらず、過体重もしくは肥満と腎結石罹患の関係を調査した研究は少ない。そこで我々は、日本人男性を対象にしてBMIと腎結石罹患の関係を調査した。

**【方法】:**

1985年に、ガス会社の労働者で、20～40歳の5,984人の男性が医学検査を受診した（ベースライン調査）。本研究はベースライン調査時において腎結石罹患歴のない4,074人の男性を対象にした。対象者は2004年4月から2005年3月の間に2回目の医学検査を受診した。対象者のBMIは1985年に測定された身長と体重から計算されて三分位に分類された。追跡期間中における腎結石罹患の有無を2回目の医学検査時に実施した自記式質問紙によって把握した。

**【結果】:**

平均19年の追跡期間中に258人が腎結石に罹患した。BMIの第1三分位群を基準にした第2および第3三分位群のハザード比（95%信頼区間）は、1.26（0.92-1.73）および1.44（1.06-1.96）であった（トレンド検定＝0.019）。潜在的交絡因子と考えられる年齢、収縮期血圧、全身持久力、喫煙量、飲酒量を調整したハザード比（95%信頼区間）は1.28 (0.93-1.76) および 1.41 (1.02-1.97)であった（トレンド検定＝0.041）。

**【結論】:**

本研究の結果は、日本人男性において、増加したBMIが腎結石罹患の危険因子であることを示唆している。

## INTRODUCTION

Kidney stones are one of the most common urological disorders in Japan. According to the Japanese urolithiasis clinical guidelines, the ratio of occurrence of upper (kidney and ureteral) and lower (bladder and urethral) urinary tract stones has been mostly stable in recent years, with upper urinary tract stones accounting for about 96% of all cases of urolithiasis.^1^ Also, the male:female ratio of patients is 2.4:1, showing a greater propensity in males. Regarding the incidence of kidney stones in Japan, upper urinary tract stone occurrence has increased markedly in recent decades.^2^ The age-adjusted annual incidence of upper urinary tract stones in 2005 was 165 per 100 000, which was more than double the incidence in 1965 (81 per 100 000).^2^ The lifetime incidence of kidney stones is not low: 15.3% in males and 6.8% in females. Moreover, the recurrence rate of kidney stones within 20 years has been reported to be about 75%.^3^

Major causes of kidney stones remain unclear in many respects, but the disease is considered to be related to a wide range of genetic, nutritional, and environmental factors. According to the 2012 National Health and Nutrition Survey in Japan, the percentage of males with a body mass index (BMI) of ≥25 is increasing (1980: 17.8%, 2012: 29.1%)^4^ in parallel to increases in the annual incidence of kidney stones. In some cross-sectional studies, body weight has been shown to be associated with a high uric acid level, low urinary pH, and increased risk of kidney stones.^5^^–^^7^ In a cohort study in the United States, higher levels of BMI were associated with increased risks of kidney stones in a dose-response relationship in men and women.^8^ The relative risk of developing kidney stones was 1.33 times higher (95% CI, 1.08–1.63) in individuals with a BMI of ≥30.0 than in those with a BMI of 21.0–22.9.^8^ In addition, in a recently reported cohort study of postmenopausal females in the United States, a strong inverse relationship was shown between physical activity and incidence of kidney stones (*P* for trend < 0.001). However, their study suggests that BMI is a predictor of kidney stones after adjusting for physical activity (*P* for trend = 0.01).^9^ There are racial differences in the prevalence of kidney stones, which is highest in whites, followed by Hispanics, blacks, and Asians.^10^ Marked differences exist in the body composition and distribution of body fat between Asians and whites,^11^^,^^12^ and to our knowledge, there has been no study of the relationship between obesity and kidney stones in an Asian population. Therefore, this study was designed to prospectively determine whether high BMI is an independent risk factor for the development of kidney stones in Japanese men.

## METHODS

### Participants

Of the workers at a natural gas company in metropolitan Tokyo, 5984 males aged 20–40 years underwent a medical examination in 1985 (baseline). Few females underwent examination (*n* = 462) and were therefore not included in this study. Of the males who underwent the baseline medical examination, 335 who had one or more of diabetes (*n* = 201), cardiovascular disease (*n* = 228), tuberculosis (*n* = 3), or gastrointestinal disease (*n* = 9) at the time of examination were excluded from this study.

In addition, those who did not undergo the exercise test for measurement of cardiorespiratory fitness as an objective marker of physical fitness in 1985 and those who reported that they had a history of kidney stones before 1985 on medical examinations performed between April 2004 and March 2005, which was the last year of the follow-up, were also excluded (*n* = 1575), leaving 4074 males for the present study.

This study was approved by the Ethics Review Board of the National Institute of Health and Nutrition.

### Measurement of BMI and potential confounders

Participants were required by the Industrial Safety and Health Act of Japan to undergo medical examinations. At the medical examination in 1985, height, body weight, and resting blood pressure were measured. Body weight was measured in light clothes and with shoes off using a scale periodically calibrated according to the law. BMI (weight in kilograms divided by the square of height in meters) was calculated from the measured height and weight. Resting blood pressure was measured using an automated sphygmomanometer while the participants were seated on a chair.

In addition, the estimated maximum oxygen uptake was measured as an index of cardiorespiratory fitness by a submaximal graded exercise test on a cycle ergometer. This test consisted of three progressively increasing submaximal 4-minute exercise stages. The heart rate was determined by ECG. The target heart rate was set at 85% of the maximum heart rate estimated from the age (220 − age), and the load was increased by 37 watts until the target heart rate or the 3rd grade was reached. The maximum oxygen uptake was estimated in each participant from the heart rate obtained during the last minute in the last grade using the nomogram of Åstrand and Ryhming^13^ and Åstrand’s age-correction factor.^14^ Drinking and smoking habits were assessed using a self-administered questionnaire.

### Diagnosis of kidney stones

In the medical examination performed between April 2004 and March 2005, participants reported whether or not they had kidney stones, as well as the duration of kidney stones, in a self-administered questionnaire, and their responses were confirmed by a nurse during a face-to-face examination. Those who answered that they had developed kidney stones after 1985 were regarded as cases for the present analysis.

### Statistical analysis

Participants were classified into tertiles according to their BMI. Baseline characteristics of the participants were compared among tertiles by one-way analysis of variance for continuous variables and the Kruskal-Wallis test for categorical variables. In this study, the relationships of BMI with the incidence of kidney stones and potential confounders, such as age, systolic blood pressure, cardiorespiratory fitness, smoking, and alcohol consumption, were analyzed using Cox proportional hazards models. We calculated hazard ratios for incidence of kidney stones according to age (10-year intervals), systolic blood pressure (10-mm Hg intervals), cardiorespiratory fitness (tertiles of maximum oxygen uptake estimated from the results of the exercise test), smoking (nonsmokers, 1–20 cigarettes per day, and ≥21 cigarettes per day), and alcohol consumption (nondrinkers, 1–45 g/day, and ≥46 g/day). In multivariable analyses, we included all potential confounders as covariates. A two-tailed *P* value less than 0.05 was considered statistically significant. Statistical analysis was performed using IBM SPSS Statistical Version 20 (IBM Japan, Tokyo, Japan).

## RESULTS

The median age of the participants at baseline was 31 (interquartile range, 28–35) years. The mean duration of the follow-up was 19 years, and 258 participants developed kidney stones during this period. Table 1 shows the baseline characteristics of the participants according to BMI categories. Participants with lower BMI tended to be younger and have lower blood pressure, while cardiorespiratory fitness tended to be higher in the low BMI group. In addition, the smoking rate was higher in the lowest BMI tertile, while alcohol consumption increased in the higher BMI groups.

**Table 1.** Baseline characteristics of participants in 1985, according to BMI groups

Characteristic	Total	1st tertile (low)	2nd tertile	3rd tertile (high)	*P* value
Participants	4074	1354	1361	1359	—
Median age, years	31 (28–35)	30 (27–34)	31 (28–35)	32 (29–36)	<0.001
Mean height, cm	169.6 (5.5)	170.1 (5.4)	169.5 (5.4)	169.2 (5.6)	<0.001
Mean weight, kg	65.8 (8.2)	58.5 (4.7)	65.3 (4.4)	73.5 (6.9)	<0.001
Mean BMI, kg/m^2^	22.9 (2.5)	20.2 (1.1)	22.7 (0.6)	25.7 (1.7)	<0.001
Mean systolic blood pressure, mm Hg	125.4 (11.6)	122.2 (11.7)	125.2 (10.9)	128.8 (11.3)	<0.001
Mean diastolic blood pressure, mm Hg	72.6 (9.0)	69.6 (8.7)	72.6 (8.6)	75.7 (8.6)	<0.001
Mean VO_2_max, mL/kg/min	41.2 (8.0)	44.1 (7.8)	41.7 (7.9)	37.8 (6.8)	<0.001
Smokers, %	66.7	68.8	65.5	65.7	0.123
Drinkers, %	69.0	63.4	70.7	73.1	<0.001

BMI, body mass index; VO_2_max, maximum oxygen uptake.

Data represented as median (interquartile range), mean (standard deviation), or %.

Table 2 shows the relationships of various potential confounders other than BMI with the incidence of kidney stones. In age-adjusted analyses, cardiorespiratory fitness and drinking habit showed weak, negative associations with the incidence of kidney stones. However, when BMI was included in the model as a covariate, the relationship between cardiorespiratory fitness and incidence of kidney stones was attenuated, but the negative association between drinking habit and incidence of kidney stones was strengthened. The incidence of kidney stones per 10 000 person-years was inversely associated with BMI (Table 3). Moreover, the high BMI group had a higher cumulative incidence rate of kidney stones during the follow-up period (Figure). Table 3 shows the crude, age- and alcohol-consumption-adjusted, and multivariable-adjusted hazard ratios of the occurrence of kidney stones by BMI tertile. In the third tertile, the multivariable adjusted hazard ratio for kidney stones was significantly (1.41 times) higher than in the first tertile (95% CI, 1.02–1.97). In addition, a significant dose-response relationship was observed between BMI and risk of kidney stones (*P* for trend = 0.041).

**Figure.**
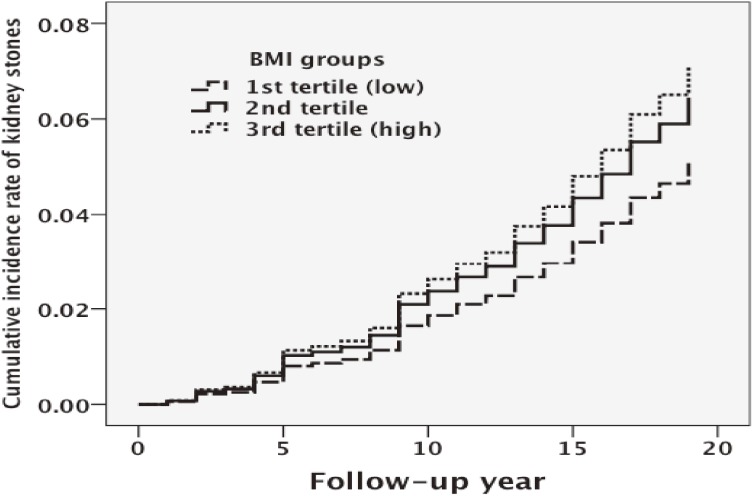
Relationship between the incidence rate of kidney stones and body mass index (BMI). The high BMI group had a higher cumulative incidence rate of kidney stones than other groups.

**Table 2.** Hazard ratios for incidence of kidney stones by potential confounders, 1985 to 2004–05

Variable	Participants	Person-years of follow-up	Incidence	Incidence (per 10 000 person-years)	Age-adjusted hazard ratio (95% CI)	*P* for trend	Multivariable^a^ Hazard Ratio (95% CI)	*P* for trend
Age								
Per 10 years	4074	77 406	258	33	—	—	0.93 (0.72–1.21)	0.601
Systolic blood pressure								
Per 10 mm Hg	4074	77 406	258	33	1.04 (0.94–1.16)	0.425	1.02 (0.91–1.14)	0.770
Cardiorespiratory fitness, mL/kg/min								
1st tertile (<37.3)	1289	24 491	90	37	1.00 (Reference)		1.00 (Reference)	
2nd tertile (37.3–43.5)	1376	26 144	91	35	0.93 (0.69–1.25)		1.02 (0.75–1.38)	
3rd tertile (>43.5)	1409	26 771	77	29	0.75 (0.55–1.03)	0.079	0.85 (0.60–1.19)	0.347
Cigarette smoking, cigarettes/day								
None	1357	25 783	86	33	1.00 (Reference)		1.00 (Reference)	
1–20	1599	30 381	89	29	0.87 (0.65–1.17)		0.89 (0.66–1.20)	
≥21	1118	21 242	83	39	1.18 (0.87–1.59)	0.324	1.20 (0.89–1.63)	0.265
Alcohol consumption, g/day								
None	1261	23 959	94	39	1.00 (Reference)		1.00 (Reference)	
1–45	2607	49 533	153	31	0.78 (0.60–1.01)		0.76 (0.59–0.99)	
≥46	206	3914	11	28	0.71 (0.38–1.33)	0.056	0.64 (0.34–1.21)	0.031

CI, confidence interval.

^a^Adjusted for all items in the table plus body mass index.

**Table 3.** Hazard ratios for incidence of kidney stones by BMI, 1985 to 2004–05

	BMI	Participants	Person-years of follow-up	Incidence	Incidence (per 10 000 person-years)	Crude hazard ratio (95% CI)	Age- and alcohol- adjusted hazard ratio (95% CI)	Multivariable^a^ hazard ratio (95% CI)
1st tertile	15.9–21.6	1354	25 726	70	27	1.00 (Reference)	1.00 (Reference)	1.00 (Reference)
2nd tertile	21.7–23.7	1361	25 859	88	34	1.26 (0.92–1.73)	1.29 (0.94–1.77)	1.28 (0.93–1.76)
3rd tertile	23.8–35.6	1359	25 821	100	39	1.44 (1.06–1.96)	1.49 (1.10–2.02)	1.41 (1.02–1.97)
*P* for trend						0.019	0.011	0.041

BMI, body mass index; CI, confidence interval.

^a^Adjusted for age, alcohol consumption, systolic blood pressure, cardiorespiratory fitness, and cigarette smoking.

## DISCUSSION

Here, we reported a cohort study designed to evaluate the relationship between overweight/obesity and kidney stones in Japanese men. A positive dose-response relationship was observed between BMI and incidence of kidney stones, and the hazard ratio of incidence of kidney stones was significantly higher in the third BMI tertile (23.8–35.6 kg/m^2^) compared with the first BMI tertile (15.9–21.6 kg/m^2^). These results suggest that BMI is a risk factor for kidney stones in Japanese men. The results of this study were similar to those of a study conducted in the United States.^8^ This large-scale cohort study of American men reported that the relative risk of kidney stones was 1.33 times (95% CI, 1.08–1.63) higher among those with BMI of ≥30.0 kg/m^2^ than in those with a BMI of 21.0–22.9 kg/m^2^. BMI differs markedly between Japanese and Americans; the proportion of the population with a BMI ≥30.0 kg/m^2^ is 4.1% in Japan but 36.5% in the United States.^15^ However, the results of the present study suggest that the risk of kidney stones is higher even with the low degree of obesity in Japanese males.

Some earlier studies reported that obesity, diabetes, and hypertension are related to kidney stones,^7^^,^^8^^,^^16^^,^^17^ and it was recently reported that kidney stones are also prevalent among patients with metabolic syndrome.^18^^,^^19^ Therefore, mechanisms involved in the etiology of lifestyle-related diseases, such as diabetes and hypertension, may also be involved in the etiology of kidney stones. One of these common mechanisms is considered to be insulin resistance. Ando et al^20^ showed that insulin and insulin resistance are correlated with an increase in the risk of self-reported kidney stones. According to De Fronzo et al,^21^ insulin suppresses calcium reabsorption by acting on the renal tubules. Shimamoto et al,^22^ using the euglycemic insulin clamp test, showed that insulin promotes the urinary and fractional excretion of calcium. In addition, there have been a few reports that obesity is related to an increase in urinary oxalate excretion and a decrease in urinary citrate excretion.^23^^,^^24^ Moreover, it was reported that urinary pH, which is a predictive factor for uric acid stones, was negatively correlated with body weight.^5^ Obesity induces insulin resistance, disturbs ammonia genesis and Na^+^/H^+^ activities, and promotes the development of ureteral stones.^25^ Thus, insulin resistance associated with obesity may increase the risk of developing calcium and uric acid-induced kidney stones.

Recently, Sorensen et al reported that physical activity may decrease the risk of incident kidney stones in postmenopausal women independent of obesity or energy intake.^9^ Since maintaining physical activity or cardiorespiratory fitness at a high level reduces the risk of lifestyle-related diseases, such as diabetes and hypertension,^26^^–^^29^ we hypothesized that cardiorespiratory fitness may also be related to the development of kidney stones. However, no significant relationship was noted between cardiorespiratory fitness and occurrence of kidney stones in the present study (Table 2). Concerning other lifestyle habits, a weak negative relationship was noted between drinking habit and kidney stones in this study (Table 2). Although the relationship between alcohol consumption and kidney stones is not well established, a negative relationship was also observed between the prevalence of kidney stones and frequency of drinking in a cross-sectional study in another Japanese population.^30^ These observations suggest that no or little drinking may be a risk factor for kidney stones. Alcohol consumption may cause an increase in urine volume and a decrease in urinary calcium concentration by suppressing antidiuretic hormone.^31^ Also, since drinking more than a certain amount of water reduces the recurrence rate of kidney stones,^32^^,^^33^ an increase in water intake associated with alcohol drinking might have an effect on the results.

A strength of the present study was that the participants were Japanese, a population in which obesity is less frequent than in Western populations. While Asian people were included in the participants of similar studies conducted in the United States, their percentage was low (approximately 3%), and whites accounted for the majority (about 80%) of the participants.^9^ To our knowledge, this is the first study to evaluate the relationship between obesity and kidney stones by following an Asian (Japanese) population, so our results may have significant implications for the prevention of kidney stones in Asians, who have relatively low mean BMI compared with whites.

However, there are some limitations in this study. First, this study had low statistical power. In a previous large study, which included 45 988 men, the distribution of BMI was divided into six groups in order to assess the risk of kidney stones across various BMI categories.^8^ However, participants of this study were only divided into tertiles because of the relatively small number of participants (*n* = 5984). Therefore, a larger cohort should be evaluated in order to obtain more robust results in Japanese men. Second, participants were male workers at a particular company, so the cohort cannot be regarded as a representative sample of Japanese men. Third, the participants of this study were all male, and a previous study showed that the relative risk of kidney stones due to obesity is higher in females than in males.^8^ We were unable to include females in the present study because of their small number. Fourth, the presence of kidney stones was self-reported at a single time point of follow-up, leading to the possibility of diagnostic and recall bias. In addition, we did not collect information on diet, and the possibility of confounding by dietary factors cannot be excluded. However, in a previous study that adjusted for dietary factors, the adjusted and unadjusted relative risks did not differ markedly, suggesting that confounding may not be a major factor.^8^ Another limitation is that BMI was based on data obtained at the baseline examination, and possible changes in BMI level were not taken into account during the follow-up period. However, not accounting for changes would only dilute the true association between BMI and the risk of developing kidney stones. Further study is necessary to investigate the relationship between changes in BMI and the incidence of kidney stones.

In conclusion, a positive dose-response relationship was observed between BMI and incidence of kidney stones. This suggests that, even in a population with relatively low BMI, higher levels of BMI are a risk factor for kidney stones in Japanese men. Future studies, such as studies that include females and studies on the influence of weight reduction on the risk of developing kidney stones, are necessary to increase understanding of the relationship between BMI and risk of kidney stones.

## ONLINE ONLY MATERIAL

Abstract in Japanese.
