# Supplementary material for: Body Mass Index and Kidney Stones: A Cohort Study of Japanese Men
Source: J Epidemiol. 2016 Mar 5;26(3):131–6. doi: 10.2188/jea.JE20150049 (PMC4773489; doi:10.2188/jea.JE20150049)
Supplement: Abstract in Japanese. [file je-26-131-s001.pdf]

BMI と腎結石の関連：日本人男性を対象にしたコホート研究

吉村英一[1,2]、澤田 亨[3]、I-Min Lee[4,5]、丸藤祐子[3]、鎌田真光[3,5]、松下宗洋[3,6]、川上諒子[3,6]、安藤亮介[7]、岡本隆史[8]、塚本浩二[8]、宮地元彦[3]、Steven N. Blair[9,10]

[1]熊本県立大学環境共生学部、[2]国立研究開発法人医薬基盤・健康・栄養研究所 栄養教育研究部、[3]国立研究開発法人医薬基盤・健康・栄養研究所 健康増進研究部、[4]ハーバード大学医学大学院、[5]ハーバード大学公衆衛生大学院、[6]早稲田大学大学院 スポーツ科学研究科、[7]名古屋市立大学大学院医学研究科、[8]東京ガス株式会社安全健康・福利室、[9]サウスカロライナ大学公衆衛生大学院運動科学科、[10]サウスカロライナ大学公衆衛生大学院疫学生物統計学科

【背景】近年、日本において腎結石罹患率は急激に増加している。腎結石の主要な原因は明確になっておらず、過体重もしくは肥満と腎結石罹患の関係を調査した研究は少ない。そこで我々は、日本人男性を対象にして BMI と腎結石罹患の関係を調査した。
【方法】1985 年に、ガス会社の労働者で、20～40 歳の 5,984 人の男性が医学検査を受診した（ベースライン調査）。本研究はベースライン調査時において腎結石罹患歴のない 4,074 人の男性を対象にした。対象者は 2004 年 4 月から 2005 年 3 月の間に 2 回目の医学検査を受診した。対象者の BMI は 1985 年に測定された身長と体重から計算されて三分位に分類された。追跡期間中における腎結石罹患の有無を 2 回目の医学検査時に実施した自記式質問紙によって把握した。
【結果】平均 19 年の追跡期間中に 258 人が腎結石に罹患した。BMI の第 1 三分位群を基準にした第 2 および第 3 三分位群のハザード比（95%信頼区間）は、1.26（0.92-1.73）および 1.44（1.06-1.96）であった（トレンド検定＝0.019）。潜在的交絡因子と考えられる年齢、収縮期血圧、全身持久力、喫煙量、飲酒量を調整したハザード比（95%信頼区間）は 1.28（0.93-1.76）および 1.41（1.02-1.97）であった（トレンド検定＝0.041）。
【結論】本研究の結果は、日本人男性において、増加した BMI が腎結石罹患の危険因子であることを示唆している。

キーワード：腎結石、BMI、全身持久力、日本人男性
